# Combining *p*‐tau217 with Other Blood Biomarkers to Enhance Prediction of Cognitive Decline: A Large Memory Clinic Cohort Study

**DOI:** 10.1002/alz70856_106988

**Published:** 2026-01-08

**Authors:** Monika Renuka Sanotra, Xuemei Zeng, Rebecca A Deek, Jeremy M. Gu, Marissa F Farinas, Michel N Nafash, Lamia Choity, Tara K Lafferty, Annie Bedison, Rocco B Mercurio, Cristy Matan, Julia K. Kofler, Dana L Tudorascu, C. Elizabeth Shaaban, Jennifer H Lingler, Tharick A Pascoal, William E Klunk, Victor L. Villemagne, Sarah B Berman, Robert Sweet, Beth E. Snitz, Milos D. Ikonomovic, Ann D Cohen, M. Ilyas Kamboh, Oscar L Lopez, Thomas K Karikari

**Affiliations:** ^1^ University of Pittsburgh, Pittsburgh, PA, USA; ^2^ University of Pittsburgh School of Medicine, Pittsburgh, PA, USA; ^3^ University of Pittsburgh Alzheimer's Disease Research Center (ADRC), Pittsburgh, PA, USA; ^4^ UPMC, Pittsburgh, PA, USA; ^5^ Department of Psychiatry, University of Pittsburgh, Pittsburgh, PA, USA; ^6^ 3501 Forbes Avenue, suite 840, Pittsburgh, PA, USA; ^7^ The University of Pittsburgh, Pittsburgh, PA, USA; ^8^ Department of Psychiatry and Neurology, University of Pittsburgh, Pittsburgh, PA, USA; ^9^ VA Pittsburgh Healthcare System, Pittsburgh, PA, USA; ^10^ University of Pittsburgh Alzheimer's Disease Research Center, Pittsburgh, PA, USA; ^11^ University of Gothenburg, Mölndal, Sweden; ^12^ Institute of Neuroscience and Physiology, Department of Psychiatry and Neurochemistry, The Sahlgrenska Academy at University of Gothenburg, Mölndal, PA, Sweden; ^13^ Institute of Neuroscience and Physiology, Department of Psychiatry and Neurochemistry, The Sahlgrenska Academy at University of Gothenburg, PA, USA; ^14^ Department of Psychiatry and Neurochemistry, Institute of Neuroscience and Physiology, The Sahlgrenska Academy, University of Gothenburg, Mölndal, Gothenburg, Sweden; ^15^ Institute of Neuroscience and Physiology, Department of Psychiatry and Neurochemistry, The Sahlgrenska Academy at University of Gothenburg, Mölndal, Sweden; ^16^ NIHR Biomedical Research Centre for Mental Health & Biomedical Research Unit for Dementia at South London & Maudsley NHS Foundation, London, United Kingdom; ^17^ Institute of Neuroscience and Physiology, University of Gothenburg, Mölndal, Sweden

## Abstract

**Background:**

Plasma *p*‐tau217 has emerged as one of the most promising biomarkers for Alzheimer's disease (AD). However, it remains largely unexplored whether integrating *p*‐tau217 with other emerging AD blood biomarkers (BBMs) will enhance its clinical performance. This research aims to address this question by analyzing a large memory clinic cohort with over three decades of longitudinal follow‐up.

**Method:**

This study utilized participants enrolled at the University of Pittsburgh Alzheimer's Disease Research Center who underwent baseline blood collection and longitudinal Clinical Dementia Rating‐Sum of Boxes (CDR‐SB) based cognitive functional assessments over thirty years. A sub‐cohort with 11C‐Pittsburgh Compound‐B (PiB) amyloid PET was used to determine the cut‐off for *p*‐tau217 (0.5471 pg/ml), according to the Youden index. Plasma levels of *p*‐tau217, *p*‐tau181, brained‐derived tau (BD‐tau), GFAP, and NfL were measured using the SIMOA assay. Statistical analysis was conducted using Cox Proportional Hazards Regression and the Kaplan‐Meier method, with events defined as an increase in CDR global score during follow‐up.

**Result:**

We included 4382 participants (57.0% female; 85.8% self‐identified non‐Hispanic White), aged 71.9 ± 9.8 years, with 2127 being non‐demented (CDR≤0.5) at baseline. High *p*‐tau217 levels were associated with a significantly faster cognitive decline, with a hazard ratio (HR) of 3.16 (CI: 2.82 – 3.53) and a median survival time of 4.0 years compared to 10.0 years for those with low *p*‐tau217. Elevated GFAP was associated with the greatest hazards when combined with high *p*‐tau217, HR 1.61 (1.41 – 1.84; *p* < 0.0001), followed by 1.46 (1.28 – 1.66; *p* < 0.0001) for NfL, 1.31 (1.14 – 1.50; *p* =  0.0002) for *p*‐tau181, and 1.29 (1.13 – 1.48; *p* =  0.0002) by BD‐tau. Among individuals with low *p*‐tau217 levels, elevated NfL was associated with the greatest hazards, HR 3.53 (2.64 – 4.72; *p* < 0.0001), followed by 1.77 (1.32 – 2.39; *p* < 0.0001) for GFAP and 1.33 (1.01 – 1.77; *p* =  0.029) for *p*‐tau181. Elevated BD‐tau in individuals with low *p*‐tau217 was not associated with greater hazards.

**Conclusion:**

Our study underscores the synergetic joint effect between *p*‐tau217 and other BBMs, and that integrating *p*‐tau217 with these BBMs can enhance tailored clinical management strategies of AD.

